# The total cost of rearing a heifer on Dutch dairy farms: calculated versus perceived cost

**DOI:** 10.1186/s13620-015-0058-x

**Published:** 2015-12-15

**Authors:** N. Mohd Nor, W. Steeneveld, T. H. J. Derkman, M. D. Verbruggen, A. G. Evers, M. H. A. de Haan, H. Hogeveen

**Affiliations:** Department of Farm Animal Health, Faculty of Veterinary Medicine, Utrecht University, P.O. Box 80151, Utrecht, 3508 TD The Netherlands; Department of Preclinical Sciences, Faculty of Veterinary Medicine, Universiti Putra Malaysia, 43400 Serdang, Selangor Malaysia; Business Economics Group, Wageningen University, P.O. Box 8130, 6706 KN Wageningen, The Netherlands; Wageningen UR Livestock Research, De Elst 1, 6708 WD Wageningen, The Netherlands

**Keywords:** Costs, Dairy, Young stock, Economics

## Abstract

**Background:**

As farmers do not often keep a record of the expenditures for rearing, an economic tool that provides insight into the cost of rearing is useful. In the Netherlands, an economic tool (Jonkos) has been developed that can be used by farmers to obtain insight into the cost of rearing on their farm. The first objective of this study is to calculate the total cost of rearing young stock in Dutch dairy herds using Jonkos. The second objective is to compare the calculated total cost of rearing with the farmers’ own estimation of the cost of rearing (the perceived cost).

**Findings:**

Information was available for 75 herds that reared their own young stock and who had used the Jonkos tool. The perceived cost of rearing young stock was only available for 36 herds. In the 75 herds, the average herd size was 100 dairy cows. The average calculated total cost of rearing a heifer was €1,790. The average perceived total cost of rearing a heifer (including labour and housing costs) was €1,030.

**Conclusion:**

Most Dutch farmers in the study underestimated the total cost of rearing. The Jonkos economic tool has the advantage that herd-specific information can be entered as input values. The output of the tool can improve the awareness of farmers about the total costs of rearing. This awareness can lead to a higher priority of young stock rearing and consequently to an improved quality of young stock rearing.

## Findings

Most Dutch dairy herds rear their own young stock to replace culled dairy cows. The total cost of rearing includes several cost factors, such as the costs of feed, housing, labour, breeding, healthcare, carcass disposal and the cost of buying heifers when not enough replacement heifers are available. Mourits et al. [1] estimated the total cost of rearing a heifer in a Dutch dairy herd (excluding labour costs) using a dynamic model. The estimates ranged between €907 and €1,134 per heifer. The cost of rearing depends on biological factors, which make it difficult to estimate. Therefore, simulation models are often used to estimate the costs [2–4]. Mohd Nor et al. [3] used a stochastic model that included uncertainty in diseases and variation in fertility and growth to estimate the total cost of rearing a heifer in a Dutch dairy herd. They estimated that the total cost of rearing a heifer was €1,567 per successfully reared heifer, with costs for feed and labour contributing the most to the total cost [3]. In the US, the average total cost of rearing a heifer (including labour costs) was estimated at $1,124 (€788) [2] and $1,808 (€1,320) [5].

In practice, farmers do not keep a record of the expenditures made for rearing their young stock. Moreover, it is hard to separate the cost of rearing young stock from the costs for the entire dairy herd enterprise. For instance, because heifers are fed the same type of feed as dairy cows, it is difficult to estimate the proportion of feed costs attributable to young stock. Because of the difficulty of knowing the costs of rearing young stock, it is expected that farmers underestimate the costs of young stock rearing and consequently do not prioritize young stock rearing enough. It is thus important for farmers to become more aware of the true cost of rearing.

An economic tool that dairy farmers can use to estimate the total cost of rearing a heifer, which is specific for their own herd, has not been developed yet. Furthermore, it is expected that farmers underestimate the costs for young stock rearing, but there is no evidence for that yet. The first objective of this study is to estimate the total cost of rearing young stock in individual Dutch dairy herds using a newly developed economic tool. The second objective is to compare the estimated total cost of rearing with the farmers’ own estimation of the cost (perceived cost of rearing). The results of this study can help farmers to become more aware of the total cost of rearing a heifer, and to prioritize young stock rearing more which will improve the quality of young stock rearing.

## Materials and methods

### Jonkos

Jonkos is an economic tool, which has been developed to calculate the cost of rearing young stock. Jonkos was developed to raise farmer awareness about the cost of rearing young stock. The estimation can be made as farm-specific or as general as the farmer wishes, by entering either general information or detailed farm information. The tool calculates both the total cost for the dairy farm and the cost per heifer. Jonkos is developed in Microsoft Excel (version 2010) and was developed as a joint collaboration between WUR Livestock research, Wageningen University Business Economics group, Faculty of Veterinary Medicine of Utrecht University and DLV (Dienst Landbouw Voorlichting). The tool Jonkos is available (in Dutch) on the internet (http://www.verantwoordeveehouderij.nl/show/JONKOS-1.htm).

**Fig. 1:**
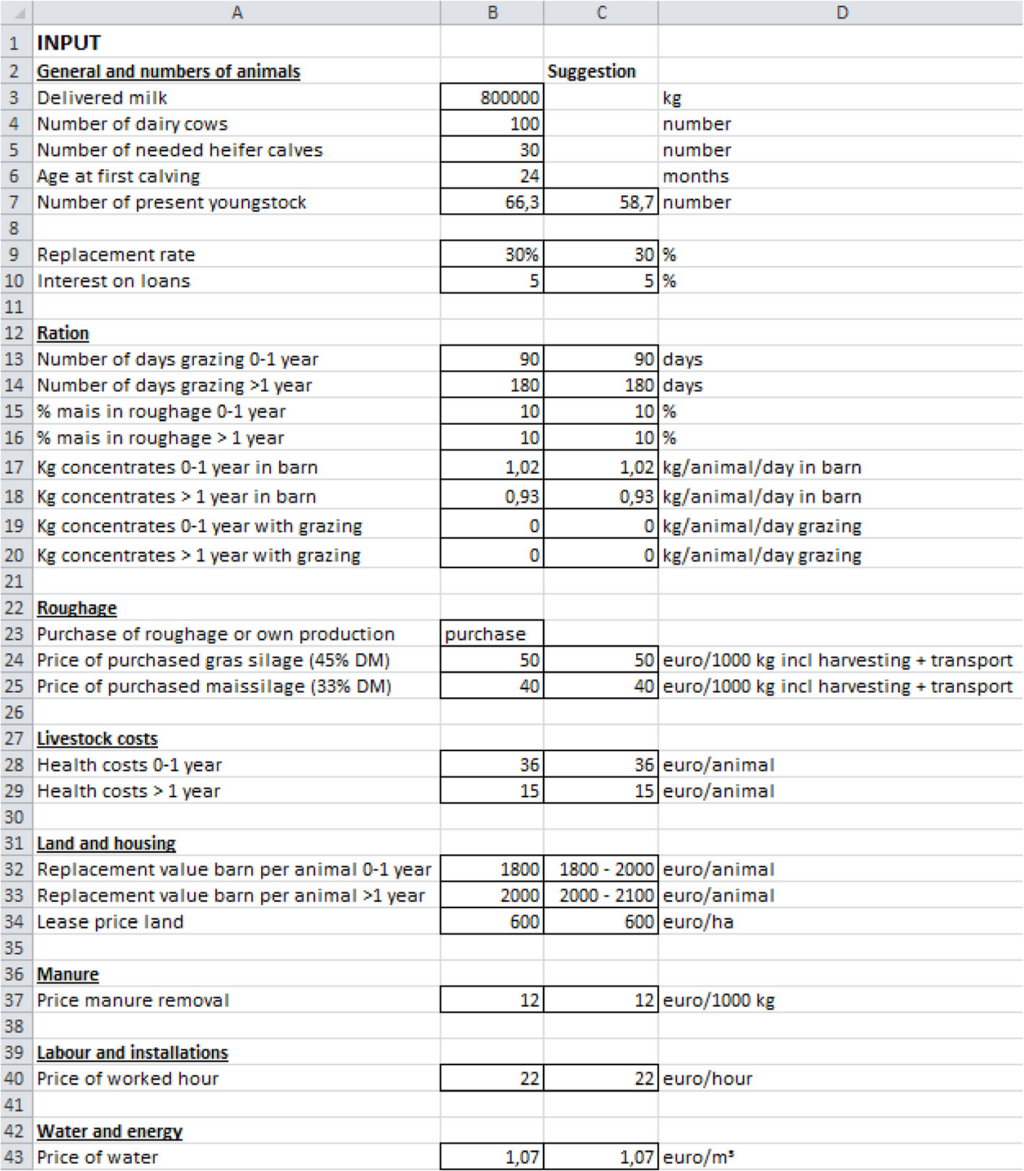
Input worksheet of the economic tool Jonkos, which calculates the total cost of rearing a heifer

**Fig. 2:**
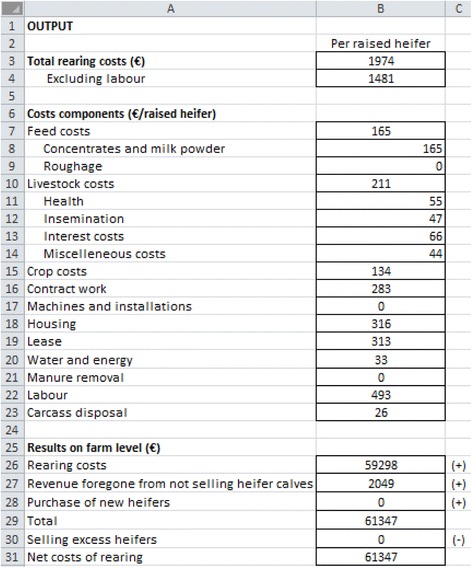
Output worksheet of the economic tool Jonkos, which calculates the total cost of rearing a heifer. A + and – means add and subtract, respectively

Jonkos consists of a main input–output worksheet, containing the most important input information grouped into eight topics (see Fig. 1). The eight topics are: general information and numbers of animals, ration, roughage, livestock, land and housing, manure, labour and installations, and water and energy. The main input–output worksheet also shows the output, including the cost of rearing young stock expressed per heifer and per herd (Fig. 2). Details of the different cost components are also presented. To calculate the net cost of rearing for a farm, the revenue foregone from not selling a two-week-old heifer calf is taken into account. Jonkos also accounts for the cost of purchasing heifers if insufficient heifers are available to replace culled dairy cows, as well as the revenue from the sale of excess heifers. In Fig. 1 the input part of the main worksheet is presented. The numbers presented in this Figure represent an average Dutch farm with 100 dairy cows, a replacement rate of 30 % and an average first calving age of 24 months. In Fig. 2 the output part of the main worksheet is presented. The numbers are the output for the farm presented in Fig. 1. The net costs of rearing for this farm are €61,347 per year.

An additional worksheet is available for each topic, where more detailed information on the topic can be entered. In the worksheet ‘general farm information and numbers’ the costs for carcass disposal, revenues foregone from not selling heifer calves and the revenues from selling excess heifers are calculated. These costs and revenues are calculated by taking into account first calving age, weaning age, mortality rate, and prices for new born calves and heifers. In the worksheet ‘ration’ the costs for milk replacer and concentrates are calculated. For the costs for milk replacer, the weaning age, the amount of milk replacer per day and the price are taken into account. Costs for concentrates were calculated by taking into account concentrates intake (included in the main input–output worksheet) and the costs for concentrates. In addition, in this worksheet the roughage requirements (maize silage, grass silage and fresh grass) are calculated (expressed in kg DM). These requirements are calculated by taking into account the intake DM per day for grass silage, maize silage and fresh grass, the percentage maize silage and grass silage in the ration (included in the main input–output worksheet) and the number of days grazing (included in the main input–output worksheet). In the worksheet ‘roughage’ the costs for crop costs, contract work, and purchase of roughage are calculated. The crop costs (separately calculated for grass and maize) are calculated by taking into the amount of land needed for young stock, and costs for seed, pesticides, fertilizer and fences. The amount of land needed is calculated by taking into account the roughage requirements and the yield per hectare. The costs for contract work (separately calculated for grass and maize) are calculated by taking into account the amount of land needed for young stock, and costs for ploughing, seeding, mowing, fertilizing, controlling weeds, and harvesting. If a farm is purchasing roughage, the roughage requirements and costs per kg DM are used to calculate the costs for roughage. In the worksheet ‘livestock’ the costs for health (included in the main input–output worksheet), insemination, shaving, bedding, interest and destruction of dead animals are calculated. In the worksheet ‘land and housing’ the costs for leasing land and housing are calculated. The costs for leasing land are calculated by taking into account the amount of land needed for young stock and the lease price of land (included in the main input–output worksheet). Housing costs includes interest, depreciation and maintenance, and were calculated by using the replacement value of the barn (included in the main input–output worksheet). The worksheet ‘manure’ includes the costs for manure removal, and were calculated by taken into account the manure production of young stock and the price for removal (included in the main input–output worksheet). Manure removal takes into account the Dutch manure policy. In the worksheet ‘labour and installations’ the costs for a milk feeding machine, an automatic concentrate feeding machine and a machine for manure removing are calculated. Interest, depreciation and maintenance costs are taken into account for these machines. The worksheet also includes the calculation of labour costs, which was calculated by taking into account the amount of hours needed for young stock and the price of labour (included in the main input–output worksheet). In the worksheet ‘water and energy’ the costs for water and energy are calculated. The costs for water was calculated by taking into account the water requirements (included drinking water and water for the milk replacer) and the price of water (included in the main input–output worksheet). Costs for energy are calculated by taking into account energy for heating milk replacer, machines and lighting, and the costs for energy.

In this way, all the costs that can be attributed to the rearing of young stock are taken into account in the Jonkos tool. All worksheets have additional columns, where Dutch averages are provided for each parameter, to assist farmers who do not know their individual farm-specific value. For instance, for the amount of DM intake for young stock older than 1 year, a value 8.8 kg DM per day [6] was suggested. The average Dutch values are based on literature [3,6] and the expertise of the developers. Economic parameters are based on Vermeij et al. [7].

Before Jonkos was used on farms, the tool was internally validated, and where necessary adapted. The effect of inputs on outputs was checked by judging whether the effect was plausible. In addition, the outcomes were also compared with literature, especially with Mohd Nor et al. [3] which was also based on Dutch circumstances.

### Available data

Using the Jonkos economic tool, the data on the cost of rearing young stock were collected from two separate studies. The first study was conducted in September 2011. In this study, 432 herds associated with the Utrecht University Large Animal practice (ULP Harmelen) were approached by email. In addition, Veterinary Health Centre ‘De Peuvers Esch’ contacted 10 of their clients by telephone to ask them if they were willing to participate in this study. In total 43 herds agreed to participate (34 from the ULP Harmelen and 9 from the De Peuvers Esch). The second study was conducted in June 2013. In this study, 177 herds associated with the Veterinary Centre Zuid-Oost Drenthe were approached by email, of which 44 herds agreed to participate.

Each study was conducted by a student from the Faculty of Veterinary Medicine (Utrecht, the Netherlands). All herds in each study were visited by one student in the last phase of his or her study. The only information provided by the student prior to the interview was that the student would come to the farm to calculate, together with the farmer, the costs of young stock rearing of the farm. No further information was provided. In the first study, the farmers were first interviewed about the perceived total cost of rearing. The farmer was asked about an estimate for the costs of rearing a heifer, and it was not allowed to look this up in recording systems or to use other sources. Subsequently, it was asked whether the estimate of the farmer included labour and housing costs. In the second study, these questions were not asked. Then, together with the student, the farmers used Jonkos to obtain an estimation of the total cost of rearing. Most entries of the farmers were based on their own estimations, and rarely recording systems or other sources were used. It was compulsory to fill in the main worksheet (see Fig. 1), and only when the farmer thought that his farm deviated from Dutch average the other worksheets were filled in. For instance, when the farm had deviating costs for insemination, mortality rate, and revenues for selling calves and heifers. Filling in Jonkos took about 20 min for each farmer. For four herds in the second study, the data was not useable as the calculations failed to save properly, leaving a total of 83 dairy herds for both studies. Only Jonkos data and the perceived heifer rearing costs were available about the farms, and only that information was used for the analyses.

The data editing was performed using Statistical Analysis System (SAS) version 9.2 (SAS Institute Inc., Cary, NC). Of the 83 herds, seven herds were specialized heifer rearing farms and one herd sold all heifer calves at 2 weeks of age. Because these eight herds differed substantially from dairy farms they were excluded from the analysis, leaving 75 dairy herds. The descriptive results for inputs and outputs were averaged across these herds. In the first study, the farmers were asked whether their perceived total cost for rearing young stock included both labour and housing costs, included labour costs only, included housing costs only, or excluded both labour and housing costs. The difference between the perceived costs and the calculated costs was calculated as the perceived cost minus the calculated cost of rearing a heifer as calculated by Jonkos, where the calculated cost was adjusted for the appropriate inclusion of labour and housing costs.

## Results and discussion

The 75 Dutch herds used in the analyses had an average herd size of 100 dairy cows (range from 40 to 220 cows). The milk production of the average herd was 811,463 kg/year. The average number of young stock present in the herds was 69 per year (Table 1). On average, the first calving age of the heifers was 25.5 months. These results show that the participating herds in the Jonkos study were bigger than the average herd size in the Netherlands [8]. The average first calving age for herds in the Jonkos study was similar to the average first calving age in the Netherlands [8].

**Table 1:** The average herd characteristics in the 75 Dutch dairy herds that rear own young stock

Variables	Average	Minimum	Maximum
Herd size	100	40	220
Milk production (kg/herd/year)	815,429	340,000	1,850,000
Milk production (kg/cow/year)	8,174	5,333	10,434
Number of present young stock per year	69	19	152

The average total cost of rearing a heifer estimated using Jonkos was €1,790, and this is €223 higher than in a previous Dutch study [3]. The additional costs not taken into account by Mohd Nor et al. [3] include costs of land ownership, crops, machinery, and contract work. These costs are associated with on-farm production of feed and were not included in the estimate of Mohd Nor et al. [3]. The total feed cost in the Jonkos study was €849 per heifer, whereas Mohd Nor et al. [3] estimated €698 per heifer. The largest cost component contributing to the total cost of rearing in the Jonkos study was the cost of labour, which was an average of €470 (26 % of the total cost). This was followed by land ownership (16 %), feed costs (15 %) and housing costs (13 %) (see Table 2). There was a huge variation across farms for different cost components. For instance, the variation in labour costs per hour was €5 and €50 (data not shown), and this caused the difference in labour costs between €129 and €1661 per reared heifer. The difference in value for labour might be caused by a difference in perception of the value of (own) labour. Also costs for land ownership varied between €0 and €609 per reared heifer. This variation is due the extreme different prices for land lease across the farms in the study. Moreover, the €0 was filled in by farmers that purchased all the feed. The huge variation in the cost components might be due to some noise in the farmers’ guesstimates. The guesstimates are however the values that the farmer use to value their young stock rearing, and are thus also used in decision making on young stock rearing.

**Table 2:** Descriptive statistics of the cost components of rearing a heifer (€), estimated using the Jonkos tool for 75 dairy herds

Cost components	Average	Minimum	Maximum
Labour	470	129	1661
Housing	236	29	499
Feed	260	74	543
Land ownership	292	0	609
Crops	67	18	173
Contract work	146	0	520
Machines and installations	84	0	516
Health	36	10	84
Insemination	34	0	76
Miscellaneous	31	2	192
Interest	57	23	107
Carcass disposal	58	0	322
Water and energy	17	0	94
Manure removal	2	0	45
Total	1790	919	3307

Previous studies indicated that feed costs accounted for 50 % to 73 % of the total cost of rearing [3,5]. In contrast, feed costs accounted for only 15 % of the total cost in the Jonkos study. This difference arises because of a different way of classifying costs. Feed costs in the Jonkos study only included the cost of milk replacer and concentrates for heifer calves. Other feed costs (production of roughage for heifers) were included in land ownership, contract work costs, and crops costs. If the same cost classification is used as the previous studies, then the actual feed costs during rearing accounted for 43 % of the total cost of rearing. This percentage is still slightly lower than previous studies [3,5].

The average net cost of rearing, estimated using Jonkos, was €54,034 per herd per year. The net cost of rearing represent all costs for rearing minus the revenues of rearing (selling excess heifers). The total cost of rearing in a 100-cow herd was previously estimated at $32,344 (€23,928, currency based on www.wisselkoers.nl) per year [4]. A comparison is difficult because of different herd systems. In the Jonkos study, the total cost of rearing (an average of €53,958 per herd per year) had the largest contribution to the net cost of rearing. Other contributing costs were the revenue foregone from not selling heifer calves at two weeks of age (an average of €6,312 per herd per year) and the cost of buying new heifers when insufficient replacement heifers are available (an average of €1,316 per herd per year). The average revenue from selling excess heifers was €7,553 per herd per year (Table 3).

**Table 3:** Costs, revenues, and net cost of rearing a heifer per herd (€), estimated using the Jonkos tool for 75 dairy herds

Variables	Average	Minimum	Maximum
Costs			
Rearing	53958	14802	129707
Revenue foregone from not selling heifer calves	6312	665	36537
Buying new replacement heifers when not enough	1316	0	35138
Revenues			
Selling excess heifers	7553	0	47614
Net cost of rearing	54034	8852	137724

In the Netherlands it is common to rear your own young stock. So farm systems where all replacement heifers are purchased do hardly exist. In fact, Dutch dairy farms keep (almost) all their newborn heifer calves to ensure enough young stock are available to replace culled dairy cows [9]. It is not known whether purchasing all replacement heifers is beneficial, and the market price of replacement heifers will highly influence this. The average price for purchasing heifers was €1015 per heifer in 2014 [10]. Because purchasing all replacement heifers is not common in the Netherlands it was decided to not include that calculation in Jonkos. Jonkos takes however into account when the farm has too little heifers available and when subsequently new heifers have to be purchased.

**Fig. 3:**
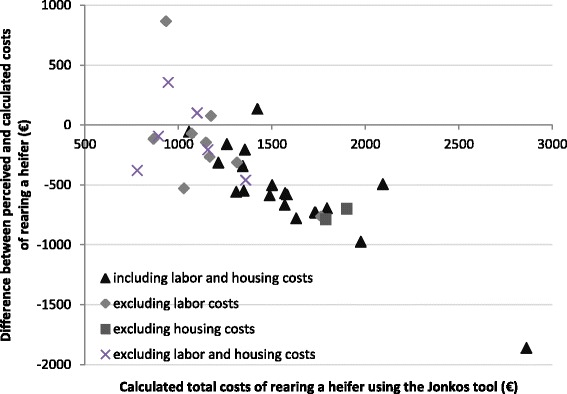
The calculated total cost of rearing a heifer using Jonkos (€) versus the difference between the perceived and calculated costs of rearing a heifer, for 37 Dutch dairy herds that rear their own young stock, classified according to the treatment of labour and housing costs

The perceived total cost of rearing a heifer in 37 herds ranged from €400 to €1,800, with an average of €1,015. The range was large because of the different treatment of labour and housing costs in these estimations. Twenty herds included labour and housing costs, six herds excluded labour and housing costs, nine herds included housing costs only, and two herds included labour costs only. For herds that included both labour and housing costs, the average perceived total cost was €1,030 (range from €750 to €1,600). For herds that excluded both labour and housing costs, the average perceived total cost was €925 (range from €400 to €1,300). For herds that included housing costs only, the average perceived total cost of rearing was €1,022 (range from €500 to €1,800) and for herds that only included labour costs, the average perceived total cost was €1,100 (range from €1000 to €1,200). The results show that most farmers (*n* = 32) underestimated the total cost of rearing a heifer (Fig. 3). The average deviation of the perceived cost from the calculated cost of rearing for herds that included labour and housing costs was -€562 (range from -€1,862 to €134). The average deviation of the cost of rearing for herds that excluded both labour and housing costs was -€115 (range from -€460 to €354). The average deviation of the cost of rearing was -€744 (range from -€788 to -€701) for herds that excluded housing costs and -€141 (range from -€764 to €866) for herds that excluded labour costs. Mourits et al. [1] found that Dutch farmers perceived the total cost of rearing to be less than Dfl 1,500 (€609) per heifer. No other study has reported the underestimation of the total cost of rearing, therefore comparison with other studies is not possible. It is important to address the underestimation of the total cost of rearing young stock, as the accuracy of the information available to farmers affects the quality of the decisions made about the rearing of young stock.

## Conclusion

The average total cost of rearing a heifer was €1,790, estimated using the economic tool Jonkos. The Jonkos tool allows a farmer to use herd-specific information to estimate the total cost of rearing young stock and the tool is easy for farmers to use. For some cost components quite detailed information must be provided, and therefore results are sometimes based on best guesses of the farmers. The average perceived total cost of rearing a heifer was €1,030, showing that Dutch farmers underestimated the true cost of rearing. By using the tool farmers can become more aware of the costs of young stock rearing. It is expected that when farmers realise that the costs for young stock rearing are much higher than estimated, that the farmers start prioritizing young stock rearing more, which will improve quality of young stock rearing. Moreover, filling in the Jonkos tool can give insight in the money that can be saved by a lower first calving age and/or retaining less young stock.
